# Associations of the Trajectories of Dietary Pattern and Hypertension: Results from the CHNS Cohort

**DOI:** 10.3390/nu18010039

**Published:** 2025-12-22

**Authors:** Hongxia Li, Zhuangyu Zhang, Die Shan, Zhiqiang Cao, Jingjing Li, Ling Liu, Yingying Ouyang, Chenrui Gong, Yuhan Tang, Ping Yao, Yi Song, Shuang Liu

**Affiliations:** 1 Department of Nutrition and Food Hygiene, School of Public Health, Tongji Medical College, Huazhong University of Science and Technology, 13 Hangkong Rd, Wuhan 430030, China; 2 Hubei Key Laboratory of Food Nutrition and Safety, School of Public Health, Tongji Medical College, Huazhong University of Science and Technology, 13 Hangkong Rd, Wuhan 430030, China; 3 Experimental Teaching Center of Preventive Medicine, School of Public Health, Tongji Medical College, Huazhong University of Science & Technology, 13 Hangkong Rd, Wuhan 430030, China; 4 Jiang’an County Health Bureau, Yibin 644299, China; 5 Institute of Health Surveillance Analysis and Protection, Hubei Provincial Center for Disease Control and Prevention, Wuhan 430079, China

**Keywords:** hypertension, trajectory of dietary patterns, group-based trajectory model

## Abstract

**Background**: Diet plays a vital role in the incidence of hypertension. Considering the complex composition and proportion of the diet and changes in eating habits, therefore, this study explores the association between the trajectory of dietary patterns and hypertension. **Methods**: A total of 1234 adults without hypertension at baseline and who provided at least three survey data from 1997 to 2018 were included. Factor analysis was used to extract dietary patterns, whose trajectories were simulated using the group-based trajectory model. The Cox proportional hazards regression model was used to analyze the association between dietary pattern trajectories and hypertension. **Results**: The factor analysis indicated four dietary patterns: the southern pattern, rice-vegetarian pattern, healthy pattern, and alcohol-meat pattern. Compared to the low-rapid rise and medium-stable groups of the southern pattern, participants in the high-stable group had a 20% reduction in the risk of hypertension (HR: 0.80; 95% CI: 0.67–0.96). The high-rapid rise group of the healthy pattern exhibited a 42% reduction in hypertension risk (HR: 0.58; 95% CI: 0.35–0.97). Conversely, individuals in the medium-high-medium subgroup of the alcohol-meat pattern displayed an increased risk of hypertension compared to those in the low-stable subgroup, with an HR of 1.48 (95% CI: 1.13, 1.94). No significant association was found between the rice-vegetarian pattern trajectory and hypertension (HR: 1.06; 95% CI: 0.85–1.34). **Conclusions**: Long-term adherence to the southern and healthy patterns might reduce the risk of hypertension, but following the alcohol-meat pattern might increase the risk of hypertension among adults in China.

## 1. Introduction

Hypertension causes structural and functional damage to multiple organs, such as the heart, brain, and kidneys, increasing the risk of various diseases, including stroke, myocardial infarction, cerebral hemorrhage, dementia, and chronic kidney disease [1]. Hypertension is a major cause of premature death worldwide [2]. A report on hypertension prevalence across WHO regions revealed that the global number of patients with hypertension increased from 650 million in 1990 to 1.4 billion in 2024, with the increase largely in low- and middle-income countries [3]. Two-thirds of adults aged 30–79 years with hypertension live in low- and middle-income countries, due to the rise in the number of older adults in these countries [3].

These findings demonstrate the severe global epidemic of hypertension, which has become one of the major causes of the global disease burden. As a developing country, the prevalence of hypertension among Chinese residents is also concerning. In recent years, the prevalence of hypertension in China has consistently increased, particularly among young and middle-aged adults. Between 1991 and 2015, the prevalence rate of hypertension in the 20–39 age group surged by 144.4% [4]. Recently, it was reported that Recent data from the China Health and Nutrition Survey displayed that the weighted prevalence of hypertension among adults aged 18 years and above in China was 27.5% in 2018, which overall trend showed significant increase in prevalence than the previous nationwide hypertension surveys conducted in 1958–1959, 1979–1980, 1991, 2002, and 2012–2015 (5.1–23.2%) [4].

Diet plays a crucial role in multiple aspects, including preventing hypertension, lowering blood pressure in hypertensive patients, and improving the efficacy of antihypertensive treatments [5,6,7,8]. A systematic review and meta-analysis incorporating 18 prospective cohort studies (451,291 participants) demonstrated that a high intake of fruits and vegetables helps reduce the risk of developing hypertension [9]. Another systematic review and meta-analysis of 23 prospective studies found that compared to the lowest intake group, the highest intake group of dairy products had a lower risk of hypertension (RR: 0.91; 95% CI: 0.86–0.95), and indicated that among different dairy subtypes, such as low-fat dairy, milk, and yogurt, the highest intake group could reduce hypertension risk by 5–12% [10]. Grillo et al. showed that long-term adherence to a reduced dietary sodium intake not only helped lower the risk of hypertension but also reduced the risk of cardiovascular diseases [11]. Although studies on the relationship between individual nutrients or food groups and hypertension have reached certain common conclusions, it is noteworthy that people typically consume multiple food groups simultaneously in dietary practices. Moreover, complex potential interactions may exist among different food groups or nutrients, making it difficult to isolate the specific effects of individual nutrients and food groups.

To address these challenges, researchers have introduced the concept of dietary pattern, aiming to comprehensively evaluate dietary intake at the holistic level [12]. Dietary pattern analysis encompasses the variety, quantity, and proportion of foods consumed in daily diets. Compared to single nutrients and food groups, dietary patterns consider the interaction and synergistic effects between foods and nutrients. Therefore, it can better explore the relationship between diet and health outcomes [13]. A community-based cross-sectional survey was conducted among adults from Northwest Ethiopia found that two types of dietary patterns: ‘westernized’ dietary pattern (meat, dairy, fast foods, alcohol, fish, sweet/sugary foods, and fruits), which is negatively correlated with hypertension, and ‘traditional’ dietary pattern (cereals, vegetables, legumes, roots/tubers, coffee, and oils), which is positively associated with hypertension [14]. Furthermore, Xu et al. identified two dietary patterns among 6348 elderly individuals aged 60 and above [15]. The “traditional pattern” was consisted of high intake of rice, pork, and vegetables, and the “modern pattern” included high intake of dairy products, fruits, cakes, and fast food, but no significant association was found between these dietary patterns and hypertension [15].

The above findings indicate that due to differences in the study population, dietary habits, and dietary assessment methods, the dietary patterns obtained through factor analysis cannot be directly compared. The conclusions regarding the relationship between dietary patterns and hypertension may not necessarily apply to the Chinese population. Importantly, most previous studies have only explored the relationship between diet and hypertension based on cross-sectional data. Even in prospective studies, dietary patterns were analyzed using only the baseline single dietary data [16,17], assuming that the dietary pattern of the participants remains constant, which fails to accurately reflect the dynamic changes in dietary patterns and the heterogeneity of the group. However, with the rapid development of the social economy and lifestyle changes, people’s dietary behaviors and dietary patterns will inevitably undergo some changes [18]. Therefore, it is necessary to conduct dietary pattern analysis based on the dietary data of the Chinese population, so as to obtain a more accurate result.

The dietary patterns are complex in the Chinese population, with both traditional and Western dietary models coexisting. Hubei Province is located in central China, and its culinary culture is rich and encompasses the characteristics of both the south and the north. Furthermore, Hubei encompasses both developed urban areas and rural regions, thereby reflecting the dietary habits and hypertension across different socioeconomic profiles. Thus, we constructed the trajectories of dietary patterns based on the Chinese dietary characteristics in Hubei Province, which provides a reference for understanding dietary patterns and hypertension in China.

## 2. Materials and Methods

### 2.1. Study Design and Population

The data for this study were sourced from the “China Health and Nutrition Survey” (CHNS) project conducted in Hubei Province. The CHNS is a large-scale longitudinal survey jointly conducted by the Chinese Center for Disease Control and Prevention and the University of North Carolina at Chapel Hill. Detailed descriptions of the survey design, technical documentation, and related materials can be found in the previous study [19]. This ongoing open prospective cohort study commenced its first wave in 1989, with follow-up surveys conducted in 1991, 1993, 1997, 2000, 2004, 2006, 2009, 2011, 2015, and 2018, totaling 10 rounds. Specifically, Hubei Province was one of the survey sites in central China, and two cities (Jingzhou and Shiyan) and four counties (Hong’an, Zaoyang, Tianmen, and Chibi) were selected. Two urban and suburban communities within cities, one community in the capital city, and three natural villages within the country were randomly chosen as investigation spots. In every survey site, 20 households were randomly selected, and all family members over two years old were invited as participants. Considering data accessibility and timeliness, this study utilized survey data from 1997 to 2018, including 8 rounds. The study was conducted in accordance with the Declaration of Helsinki and approved by the Ethics Committees of the National Institute for Nutrition and Health at the Chinese Center for Disease Control and Prevention in Beijing, China (No. 2018-004). All participants provided written informed consent.

**Figure 1:**
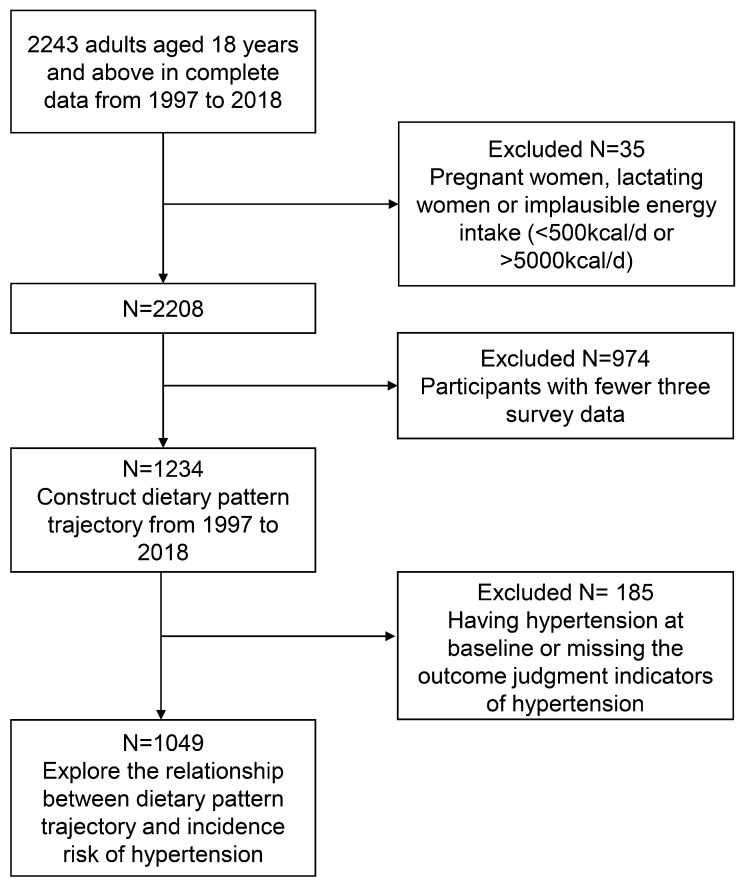
Flow chart of inclusion and exclusion criteria.

We included adults aged 18 years or older during the survey period from 1997 to 2018, and those who had complete dietary survey data available. We excluded pregnant and lactating women and individuals with excessively high or low energy intakes (<500 kcal/d or >5000 kcal/d) from the general population. Among the remaining 2208 participants, we further excluded 974 subjects who had fewer than three rounds of survey data. The baseline characteristics of the included and excluded populations were largely similar, except for the place of residence and the physical activity level. 1234 participants were involved in constructing the trajectory of dietary patterns for 1997–2018. We further excluded those with missing data for hypertension outcome assessment and those with baseline hypertension. Finally, 1049 subjects were included to analyze the association between the trajectory of dietary patterns and the incidence of hypertension (Figure 1). In the primary analysis, missing continuous variables were imputed using the median, and missing categorical variables were addressed by generating missing indicator variables.

### 2.2. Definition of Hypertension

The measurement is conducted using a calibrated mercury sphygmomanometer (with a graduation value of 2 mmHg). Before the measurement, the participants were required to rest for at least 15 min. During the measurement, ensure that the upper arm of the participant is at the same level as the heart. Each participant underwent three repeated measurements of the right arm following standard procedures. The average of the three systolic blood pressure (SBP) and diastolic blood pressure (DBP) measurements was recorded as the final blood pressure value. Hypertension was defined as SBP ≥ 140 mmHg and/or DBP ≥ 90 mmHg and/or a previous physician diagnosis of hypertension and/or current use of antihypertensive medication, according to the 2018 Chinese Guidelines for Prevention and Treatment of Hypertension [20]. New-onset hypertension refers to those participants who did not have hypertension at baseline but met the diagnostic criteria for hypertension in the subsequent follow-up. If there are multiple records or inconsistent records regarding the diagnosis of hypertension, to reduce the recall bias, only the first record that meets the diagnostic criteria will be used to determine the new-onset hypertension. The onset time was defined as the midpoint between the round survey year when the participants were first diagnosed with hypertension and the most recent survey year prior to that round [21].

### 2.3. Diet Intake

At the individual level, the 24 h dietary recall method over three consecutive days (including two weekdays and one weekend day) was employed to collect individual-level dietary intake data. Dietary data were obtained through face-to-face interviews to record all food types and quantities consumed by the participants during the 24 h. At the household level, a weighing and recording method was used to collect data on the intake of various condiments during the same three-day period. In each round of data collection, professional training was provided to the investigators. Uniform and standardized questionnaires were used, and clear instructions on the filling of each piece of information were given. A detailed survey guide was also compiled. The accurate and reliable weighing tools were also provided to the participants, and the investigators guided the participants on how to use the weighing tools correctly and how to record the data properly. During the investigation process, investigators repeatedly asked the participants to recall their diet the previous day accurately and comprehensively. During data processing, double-entry was conducted by two investigators to ensure the accuracy of the data.

### 2.4. Covariates

In each round of the survey, we obtained data on covariates such as social demographics, lifestyle, and disease status through questionnaires, physical examinations, and biochemical indicator tests. Information on age, sex, residence area, education level, marital status, smoking status, alcohol consumption, physical activity level, and history of diabetes was collected using a questionnaire. Body mass index (BMI) was calculated as weight (kg) divided by the square of height (m^2^). The definition of BMI change is the difference between the BMI of the subject in the last survey and the BMI in the baseline. The definition of change in sodium intake is the difference between the sodium intake of the subject in the last survey and the sodium intake in the baseline. Diabetes was defined as being diagnosed with diabetes by a physician and/or receiving special hypoglycemic treatment and/or FBG ≥ 7.0 mmol/L and/or HbA1c ≥ 6.5%.

### 2.5. Statistical Analysis

Frequencies (percentages) were used to describe categorical variables, and continuous variables were described using the mean ± standard deviation (SD). Continuous variables used the two-sample independent t-test, and categorical variables used the Chi-squared test to compare the differences between groups.

Exploratory factor analysis was employed to construct the dietary patterns. A total of 17 food groups were included in the analysis in this study (including rice, other grains, fruits, bean products, vegetables, pickled vegetables, wheat products, dairy products, eggs, nuts, aquatic products, poultry, pork, other livestock meats, offal, alcoholic beverages, and pastry products). If the Kaiser-Meyer-Olkin (KMO) value was ≥0.5 and Bartlett’s test was statistically significant (*p* < 0.05), it indicated that exploratory factor analysis was appropriate for the dietary data. Next, the number of major dietary patterns to retain in the study was determined based on scree plot inflection points, eigenvalues ≥ 1, and interpretability of factor characteristics. In this study, we calculated the factor loadings after varimax orthogonal rotation. A factor loading absolute value ≥ 0.25 was used as the criterion for identifying major contributing food groups. Finally, we used the regression method to estimate the factor scores for each dietary pattern, calculated as the sum of the products of the standardized food group intakes and their corresponding rotated factor loadings. A positive factor score indicates a higher-than-average adherence to the dietary pattern, whereas a negative score indicates lower-than-average adherence. Thus, the likelihood of an individual following a specific dietary pattern can be inferred from the factor score, with higher scores indicating greater adherence.

The group-based trajectory model (GBTM) was used to fit and construct dietary pattern change trajectories among adult residents of Hubei Province from 1997 to 2018. First, select an appropriate fitting model based on the data types of the research variables. The study chose a censored normal distribution because the dietary pattern factor score is a continuous variable. Second, the optimal number of trajectory groups and curve shapes of each trajectory group was determined. This study sequentially fitted one to four trajectory groups, modeling each group’s shape distribution using cubic, quadratic, and linear models. The criteria for evaluating the specific model encompassed: (1) ensuring the simplicity of the model; (2) evaluating model fit using the Bayesian Information Criterion (BIC), where absolute BIC values closer to 0 signify a superior model fit; (3) employing an average posterior probability (AvePP) exceeding 0.7 as the threshold for grouping; (4) Entropy is an indicator used to measure the accuracy of a model’s classification. Its value ranges from 0 to 1. Generally, the closer the value is to 1, the better the model’s fitting effect is [22].

A multivariable-adjusted Cox proportional hazards regression model was employed to investigate the association between different dietary pattern trajectories and the risk of hypertension, calculating the corresponding HRs and 95% CIs. The baseline was defined as the survey year when each subject first entered the investigation and had complete dietary data. The follow-up period was defined as non-hypertensive individuals spanned from the baseline to the year of the last follow-up; for hypertensive individuals, it spanned from the baseline to the year of the first hypertension diagnosis. Multiple models were used to adjust for potential confounding factors. We calculated the incidence rate of hypertension by dividing the number of newly diagnosed hypertension cases during follow-up by the person-years. In all models, the reference group comprised individuals with the lowest propensity for the respective dietary patterns. Model 1 was adjusted for age (years, continuous), sex (male or female), residence (rural or urban), education level (primary school or below, middle school, high school or above), and marital status (married or unmarried). Model 2 was further adjusted for smoking status (yes or no), alcohol consumption (yes or no), physical activity level (light, moderate, or vigorous), and total energy intake (kcal/day, continuous). Model 3 was additionally adjusted for diabetes status (yes or no), baseline BMI (kg/m^2^, continuous), and BMI change (kg/m^2^, continuous). Model 4 was further adjusted for changes in sodium intake (g/day, continuous), and Model 4 was used as the full model in this study.

### 2.6. Sensitivity Analysis

To examine the robustness of the main findings, this study conducted the following sensitivity analyses: First, because participants may alter their dietary behaviors in compliance with medical advice after being diagnosed with chronic diseases such as diabetes, stroke, or myocardial infarction, this study repeated the regression analysis after excluding populations with baseline diabetes, stroke, or myocardial infarction. Second, to assess the potential impact of missing covariates on the primary outcomes, this study employed multiple imputation methods to handle missing values on one hand, and on the other hand, repeated the main analysis after excluding study populations with missing values in the covariates included in the full model (Model 4).

We used SAS 9.4 (SAS Institute Inc., Cary, NC, USA), State17.0, and R 4.1.1 (R Development Core Team, Vienna, Austria) for statistical analyses. All tests were bilateral, and statistical significance was set at *p* < 0.05.

## 3. Results

### 3.1. Extraction and Analysis of Dietary Patterns

Among the 2208 individuals, 1234 with at least three complete dietary records were included in the model of dietary pattern change trajectories. Factor analysis was employed to extract the main dietary patterns (KMO = 0.59, *p* < 0.001 for Bartlett’s test of sphericity). We obtained four patterns based on the inflection point of the scree plot, eigenvalues ≥ 1, and the interpretability of dietary pattern factors, which collectively explained 34.47% of the total variance (Supplementary Figure S1). The eigenvalues were 1.861, 1.663, 1.202 and 1.134, and the principal component contribution rates were 10.9%, 9.8%, 7.1% and 6.7%, respectively.

Absolute factor loadings of 0.25 or higher as the main contributing food components, which were then named according to the dietary characteristics. As shown in Table 1, pattern 1 was characterized by a higher consumption of rice (0.405), aquatic products (0.478), and pork (0.275), along with a lower intake of wheat (−0.769) and other grains (−0.402). This reflects the typical eating habits of southern Chinese residents, who primarily consume rice as their staple food. Hence, it is named the “southern pattern”. In pattern 2, vegetables (0.731), rice (0.667), and pickled vegetables (0.406) exhibited high factor loadings, indicating the presence of the “rice-vegetarian pattern” within the study population. Pattern 3 included milk (0.699), fruits (0.668), nuts (0.342), poultry (0.305), and eggs (0.277), which also demonstrated elevated factor loadings, reflecting a greater consumption of these foods and thus termed the “healthy pattern.” In pattern 4, alcoholic beverages (0.633), organ meats (0.474), pork (0.389), other livestock meats (0.372), soy products (0.370), poultry (0.342), and aquatic products (0.307) exhibited high factor loadings, representing the “alcohol-meat pattern” in the study population, which is characterized by increased alcohol consumption accompanied by a variety of meat products (Table 1).

### 3.2. Construction of Dietary Pattern Trajectories

**Figure 2:**
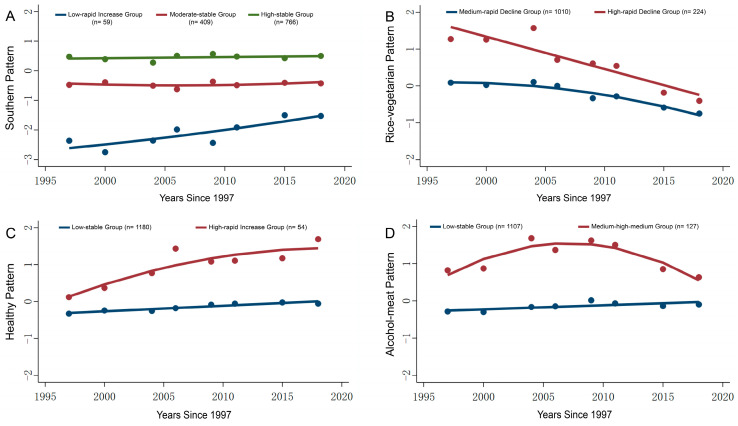
Trajectories of different dietary patterns among the study population from 1997 to 2018. The X axis represents the survey year, while the Y axis indicates the dietary pattern factor scores for different dietary patterns. Dots represents the factor scores of the trajectories of different dietary patterns in different years. Positive scores indicate that the participants have a higher tendency towards this dietary pattern compared to the average level, while negative scores indicate a lower tendency. The higher the dietary pattern factor score, the greater the tendency towards this dietary pattern. (**A**) The trajectories of southern pattern. (**B**) The trajectories of rice-vegetarian pattern. (**C**) The trajectories of healthy pattern. (**D**) The trajectories of alcohol-meat pattern.

This study utilized the GBTM to construct trajectories representing various dietary patterns. The dietary pattern score was a continuous variable, approximately following a normal distribution (Supplementary Figure S2), so a truncated normal model was selected for fitting. As shown in Supplementary Table S1, the AvePP of each trajectory group was greater than 0.7, and BIC and entropy also indicated that the models fit well. The parameters of the multiple fitting processes of each dietary pattern trajectory are shown in Supplementary Table S2. Following the trajectory fitting procedures with different combinations, the southern pattern ultimately yielded three optimal trajectories, while the rice-vegetarian pattern, healthy pattern, and alcohol-meat pattern each produced two optimal trajectories (Figure 2).

In the southern pattern (Figure 2A), the low-rapid increase group (*n* = 59, 4.8%) exhibited a relatively lower preference for this pattern compared to other groups in 1997; however, it demonstrated a consistently rapid increase in preference in subsequent survey years. The moderate-stable group (*n* = 409, 33.1%) displayed a moderate preference for this pattern at baseline in 1997, with minimal fluctuations in later surveys. Conversely, the high-stable group (*n* = 766, 62.1%) showed a high preference for this pattern at baseline in 1997, maintaining consistently higher and more stable preference than the other two groups throughout the subsequent surveys.

In the rice-vegetarian pattern, the medium-rapid decline group (*n* = 1010, 81.8%) exhibited a moderate average preference for this pattern in 1997, followed by a rapid decline in subsequent surveys. Conversely, the high-rapid decline group (*n* = 224, 18.2%) demonstrated a strong preference for this pattern in 1997, showing a similarly rapid declining trend in the following survey years, while maintaining an overall higher preference than the medium-rapid decline group (Figure 2B).

In the healthy pattern (Figure 2C), the low-stable group (*n* = 1180, 95.6%) exhibited a low level of preference for this pattern in 1997, with relatively stable changes observed in subsequent surveys. In contrast, the high-rapid increase group (*n* = 54, 4.4%) demonstrated a high level of preference for this pattern at baseline in 1997, showing a rapidly increasing trend in subsequent surveys while consistently maintaining higher preference levels than the low-stable group.

In the alcohol-meat pattern, the low-stable group (*n* = 1107, 89.7%) exhibited a low preference for this pattern in 1997, with minimal changes observed in subsequent surveys. The medium-high-medium group (*n* = 127, 10.3%) demonstrated a moderate preference for this pattern at baseline in 1997, reached a higher level between 2004 and 2009, and subsequently declined back to a moderate level after 2011, but the overall preference for the pattern remained higher than that of the low-stable group (Figure 2D).

### 3.3. Baseline Characteristics of the Study Population Based on Dietary Pattern Trajectory Grouping

The current study enrolled 1049 participants free of hypertension at baseline, comprising 510 males (48.6%) and 539 females (51.4%). The baseline characteristics of the study subjects, categorized by different dietary pattern trajectories, are presented in Table 2.

As indicated in Table 2, due to the small sample size of the low-rapid rise group in the southern pattern (*n* = 46), the low-rapid rise group and the medium-stable group were combined for subsequent analysis. The results revealed that, compared to the combined low-rapid rise and medium-stable group, the high-stable group was younger (39.5 years vs. 41.7 years), had a lower proportion of rural residents (66.4% vs. 75.3%), lower alcohol consumption (38.0% vs. 48.1%), and lower levels of heavy physical activity (46.7% vs. 57.1%). Conversely, the proportion of individuals with a high school education or above was higher in the high-stable group (27.6% vs. 18.0%).

Compared to the medium-rapid decline group in the rice-vegetarian pattern, the high-rapid decline group was more likely to be male (62.3% vs. 45.3%), reside in rural areas (95.1% vs. 63.6%), and engage in heavy physical activity (85.8% vs. 42.0%). However, the proportion of individuals with a high school education or above was lower in the high-rapid decline group (12.7% vs. 26.7%).

In the healthy pattern, compared to the low-stable group, the high-rapid rise group was older (44.5 years vs. 40.1 years), less likely to be rural residents (15.2% vs. 72.2%), and had a higher proportion of individuals with a high school education or above (56.5% vs. 22.5%).

Compared to the low-stable group in the alcohol-meat pattern, the medium-high-medium group had higher proportions of males (78.2% vs. 45.2%), individuals with a high school education or above (33.6% vs. 22.9%), smoking (51.8% vs. 28.6%), and alcohol consumption (71.8% vs. 38.2%).

### 3.4. Dietary Characteristics of the Study Population Based on Dietary Pattern Trajectory Grouping

The dietary characteristics of the study population, categorized according to different dietary pattern trajectories, were presented in Supplementary Tables S3–S6. In the southern pattern, compared to the low-rapid rise and medium-stable groups, the high-stable group had a higher intake of dark-colored vegetables, legumes, aquatic products, eggs, red meat, and fats, while their intake of grains, whole grains, tubers, sodium, energy, and carbohydrates was lower (Supplementary Table S3). In the rice-vegetarian pattern, the high-rapid decline group exhibited a higher intake of grains, vegetables, dark-colored vegetables, aquatic products, sodium, energy, protein, and carbohydrates compared to the medium-rapid rise group, while showing lower intake levels of whole grains, legumes, eggs, red meat, and fats (Supplementary Table S4). In the healthy dietary pattern, the high-rapid rise group displayed higher intake levels of fruits, legumes, aquatic products, poultry, eggs, nuts, red meat, protein, and fats compared to the low-stable group, while exhibiting lower intake levels of grains, sodium, and carbohydrates (Supplementary Table S5). In the alcohol-meat pattern, the medium-high-medium group showed higher intake levels of legumes, aquatic products, poultry, red meat, sodium, energy, protein, and fats compared to the low-stable group, while displaying lower intake levels of grains (Supplementary Table S6).

### 3.5. Association of Dietary Pattern Trajectories and Hypertension Incidence Risk

This study included 1049 participants with a cumulative follow-up duration of 11,725.5 person-years and a median follow-up time of 10.5 years (IQR: 7.0–16.0 years). During the follow-up period, a total of 529 individuals were diagnosed with incident hypertension. The results of the association between different dietary pattern trajectories and hypertension risk are presented in Table 3.

In the southern pattern, due to the small sample size in the low-rapid increase group, the low-rapid increase and medium-stable groups were combined to serve as the reference group for analysis. After adjustment, individuals in the high-stable group exhibited a lower risk of hypertension compared to the combined low-rapid increase and medium-stable group (HR: 0.80; 95% CI: 0.67–0.96). No association was found between the rice-vegetarian pattern trajectory and hypertension risk (HR: 1.06; 95% CI: 0.85–1.34). In the healthy pattern, individuals in the high-rapid increase group showed a negative correlation with the risk of hypertension (HR: 0.58; 95% CI: 0.35–0.97). In the alcohol-meat pattern, compared to the low-stable group, individuals in the medium-high-medium group were positively associated with hypertension risk after adjustment (HR: 1.48; 95% CI: 1.13–1.94).

### 3.6. Sensitivity Analysis

After excluding participants with diabetes, stroke, and myocardial infarction at baseline, the association between dietary pattern trajectory and hypertension remained robust among the remaining 1026 individuals. Following adjustment, the high-stable group in the southern pattern and the high-rapid increase group in the healthy pattern remained protective factors against hypertension, with HRs and 95% CIs of 0.81 (0.67–0.97) and 0.54 (0.31–0.91), respectively (Supplementary Table S7). Conversely, the medium-high-medium group in the alcohol-meat pattern remained a risk factor for hypertension (HR: 1.48; 95% CI: 1.13–1.93). No association was found between the high-rapid decline group in the rice-vegetarian pattern and hypertension risk (HR: 1.04; 95% CI: 0.83–1.32).

After imputing missing covariate values using multiple imputation, as shown in the Supplementary Table S7, the high-stable group in the southern pattern and the high-rapid increase group in the healthy pattern were negatively associated with hypertension risks, with HRs and 95% CIs of 0.83 (0.69–0.99) and 0.60 (0.36–0.99), respectively. Conversely, the medium-high-medium group in the alcohol-meat pattern was positively associated with hypertension risk (HR: 1.45; 95% CI: 1.11–1.90). No significant association was observed between the high-rapid decline group in the rice-vegetarian pattern and hypertension risk (HR: 1.07; 95% CI: 0.85–1.34), which is consistent with the aforementioned findings.

After excluding participants with missing covariate data, the relationship between dietary pattern trajectories and hypertension risk was analyzed among the remaining 807 individuals. As shown in the Supplementary Table S7, the high-stable group in the southern pattern was associated with a 23% reduction in hypertension risk (HR: 0.77; 95% CI: 0.62–0.95), while the high-rapid increase group in the healthy pattern was associated with a 42% reduction in hypertension risk (HR: 0.58; 95% CI: 0.34–0.98). Conversely, the medium-high-medium group in the alcohol-meat pattern significantly increased hypertension risk (HR: 1.37; 95% CI: 1.02–1.84). No significant association was found between the rice-vegetarian pattern and hypertension risk.

## 4. Discussion

The results of factor analysis indicated the presence of four distinct dietary patterns: the southern pattern, rice-vegetarian pattern, healthy pattern, and the alcohol-meat pattern. Each dietary pattern exhibited a unique trajectory. The high-stable trajectory in the southern pattern and the high-rapidly increasing trajectory in the healthy pattern were negatively correlated with the risk of hypertension, whereas the medium-high-medium trajectory in the alcohol-meat pattern was positively correlated with the risk of hypertension. No significant association was observed between the rice-vegetarian pattern trajectory and the risk of hypertension.

The variance contribution rates of these four dietary patterns were 10.9%, 9.8%, 7.1%, and 6.7%, respectively, resulting in a cumulative variance contribution rate reaching 34.5%. This cumulative rate was higher than the variance contribution rates reported by Wang et al., which were 10.3%, 6.5%, 6.0%, and 5.6% (the cumulative variance contribution rate is 28.4%), based on four dietary patterns extracted from 2718 adult residents in Suzhou, China [23]. Moreover, the cumulative variance contribution rate in this study was also higher than that of a study conducted in Australia (21.5%) [24].

This study constructed trajectory models for dietary pattern factor scores using the GBTM and identified three trajectories for the southern pattern, while each of the other dietary patterns exhibited two trajectories. Similarly, a study involving 4360 Australians applied the GBTM to fit dietary pattern trajectories from ages 14 to 22 and found two trajectories for each dietary pattern [25]. Another cohort study from Finland, which included 1007 participants, also identified three distinct trajectories for each dietary pattern over a 31-year follow-up period [26]. After adjusting for multiple confounding factors, they found that compared to the low-stable trajectory of the red meat pattern, belonging to either the ascending trajectory group or the moderate-stable trajectory group was positively associated with impaired fasting glucose [26].

The results of this study indicated that compared to the low-rapid rise group and medium-stable group, individuals in the high-stable group of the southern pattern had a lower risk of hypertension (HR: 0.80; 95% CI: 0.67–0.96). Higher dietary pattern scores represent greater adherence to the pattern. The findings suggested that long-term adherence to the southern pattern (characterized by a higher consumption of rice, aquatic products, and pork, along with a lower intake of wheat and other cereals) may reduce the risk of hypertension. A study based on the CHNS from 1991 to 2018, which included 15,929 adults, identified the southern pattern (characterized by high intake of rice, vegetables, pork, fish, and seafood) as the predominant dietary pattern among the Chinese [17]. Linear regression and logistic regression models were utilized to assess the relationship between the southern pattern and SBP, DBP, and hypertension risk; however, no significant association was observed among them [17]. Notably, the previous study only used dietary data collected at a single time point at baseline, whereas our study employed the GBTM approach to fit southern pattern trajectories with eight rounds of data. The approach better reflected the dynamic changes in the southern pattern during long-term follow-up and provided stronger evidence for investigating the association between southern pattern trajectories and hypertension risk from a long-term, dynamic perspective.

The findings of this study revealed that compared to the low-stable group, participants in the high-rapid increase group of the health pattern exhibited a 43% reduced risk of hypertension (HR: 0.57; 95% CI: 0.34–0.96), indicating that long-term adherence to a healthy pattern might contribute to hypertension prevention. The healthy pattern identified in this study was primarily characterized by high consumption of dairy products, fruits, nuts, eggs, and poultry, suggesting that the protective effects against hypertension may stem from the combined benefits of these food groups. A systematic review and meta-analysis incorporating 28 prospective studies demonstrated that a daily intake of 100 g of fruits and 200 g of dairy products was associated with 3–5% reductions in hypertension risk, respectively [27].

Compared to the low-stable group, long-term adherence to the alcohol-meat pattern (medium-high-medium trajectory group) was associated with higher proportions of males (78.2% vs. 45.2%), smokers (51.8% vs. 28.6%), and alcohol consumers (71.8% vs. 38.2%). Some studies have indicated that the association between alcohol consumption and increased risk of hypertension was only observed in males, while no correlation was found in females [28,29]. A systematic review and meta-analysis, which included 36 trials, showed that reducing alcohol intake was not associated with blood pressure changes among individuals consuming no more than two drinks per day (≤24 g of pure alcohol). However, in heavier drinkers, particularly those consuming more than six drinks per day (≥72 g of pure alcohol), reducing alcohol intake effectively lowered blood pressure levels [30]. The potential protective effect of moderate alcohol consumption on vascular function remains controversial, and the relationship between alcohol consumption and hypertension is still inconclusive [31,32]. Nevertheless, the findings of this study demonstrate a positive correlation between long-term alcohol consumption and the risk of hypertension. Therefore, it is essential to limit alcohol intake among long-term drinkers.

No association was found between the rice-vegetarian pattern and the risk of hypertension, indicating that long-term adherence to a rice-based vegetarian diet might not be related to the risk of developing hypertension. The dietary characteristics of the rice-vegetarian pattern in this study included a high intake of rice, vegetables, and pickled vegetables. Wang and colleagues conducted a study involving 2718 adults from communities in eastern China, which similarly reported no correlation between a “rice-vegetable pattern” and hypertension [23,28]. Likewise, Chen et al. did not observe a significant effect of a rice-vegetable pattern on blood pressure in 6849 adults aged 21–70 from southwestern China [33].

A prospective cohort study conducted in Iran involving 10,047 adults aged 35 to 65 found that individuals in the third quartile of a plant-based dietary pattern had a 30% lower risk of hypertension incidence compared to the first quartile (HR: 0.70; 95% CI: 0.53–0.94). Notably, the plant-based dietary pattern in this study was comprehensive, emphasizing leafy vegetables, fresh fruits, legumes, and whole grains. The protective effect against hypertension was likely attributable to the combined influence of various health-promoting plant-based foods, rather than vegetables alone [34].

Since the beginning of the 21st century (from 2001 to 2012), the economy of China has grown rapidly, the process of urbanization has accelerated, and disposable income has increased [35]. The dietary patterns were gradually shifting towards higher energy, higher fat, higher salt content, and increased alcohol consumption [36,37]. The consumption of fast food and ultra-processed foods in cities has increased, while the intake of vegetables and fruits is relatively insufficient [38]. Consistent with our findings, the trajectory of the rice-vegetarian pattern has gradually declined, while that of the meat-and-beverage diet has shown a rapid increase from 1997 to 2012. After 2012, China entered a new era of high-quality economic development. The *Dietary Guidelines for Chinese Residents* were revised several times (2007, 2016, and 2022), and more extensive health education was carried out [39]. Therefore, health awareness of residents has significantly increased, which will also affect the dietary pattern. In this study, the trajectory of the rice-vegetarian pattern has shown a downward trend, while the trajectory of the alcohol-meat pattern has shifted from an upward trend to a significant downward trend. The trajectory of the healthy pattern has been showing an increasing trend, and the trajectory of the southern pattern, a traditional dietary pattern, has remained stable. Although there is evidence indicating that the dietary quality of Chinese residents has significantly improved [40], however, there are still new challenges related to the risk of hypertension that need to be vigilant about, such as reduced physical activity and increased mental health problems (anxiety, depression), etc. [41].

The findings of this study provide valuable insights into the association between dietary pattern trajectories and hypertension risk in the Chinese population. The long-term follow-up and multiple dietary surveys enhance the reliability of our findings. However, several limitations should be acknowledged. Firstly, dietary data were collected using the 24 h dietary recall method, which may be subject to recall bias. Despite rigorous quality control and standardized training for all staff, and the use of a 3-day consecutive recall for individual-level data and food weighing for household-level data to improve accuracy, recall bias is undoubtedly difficult to avoid. Secondly, although the results were consistent with the primary analysis in several sensitivity analyses, and adjustments were made for some potential covariates that could influence the relationship between dietary pattern trajectories and hypertension, measurement errors or unmeasured confounders may still affect our findings. For instance, family history of hypertension could not be included as a covariate due to incomplete data. Thirdly, participants with hyperlipidemia or hyperuricemia could not be excluded because lipid data were only available in 2009 and 2015, but not collected at baseline. Fourthly, the participants in our study were selected from Hubei province, China, which may limit the generalizability of the findings to other populations. Finally, individuals were grouped into different dietary pattern trajectories based on posterior probabilities by GBTM, which involves certain uncertainties. Because some individuals may be at the boundaries of different trajectory groups, their classification may not be clear. It could potentially affect the accurate assessment of an individual’s dietary pattern and the formulation of intervention measures in practical applications. Therefore, further study involving diverse populations from different regions and ethnicities is needed to validate the association between dietary pattern trajectories and hypertension.

## 5. Conclusions

Long-term adherence to the southern pattern and the healthy pattern was negatively associated with the risk of hypertension, whereas persistent adherence to the alcohol-meat pattern may increase the risk of hypertension, particularly in males.

## Figures and Tables

**Table 1:** The factor loadings of dietary patterns from 1997 to 2018.

Food Species	Southern Pattern	Rice-Vegetarian Pattern	Healthy Pattern	Alcohol-Meat Pattern
Wheat products	**−0.769**	−0.140	−0.023	0.135
Aquatic products	**0.478**	0.125	0.092	**0.307**
Other grains	**−0.402**	0.016	−0.044	−0.093
Vegetables	−0.027	**0.731**	0.054	−0.013
Rice	**0.405**	**0.667**	−0.150	−0.248
Pastry products	**0.435**	**−0.457**	−0.043	0.072
Pickled vegetables	0.047	**0.406**	−0.068	0.097
Dairy products	−0.083	0.006	**0.699**	−0.116
Fruits	0.132	−0.141	**0.668**	0.058
Nuts	0.018	0.179	**0.342**	0.144
Eggs	0.180	−0.125	**0.277**	0.020
Alcoholic beverages	−0.121	0.193	−0.091	**0.633**
Offal	0.048	−0.090	−0.134	**0.474**
Pork	**0.275**	−0.065	0.245	**0.389**
Other livestock meats	0.063	−0.083	0.237	**0.372**
Bean products	0.184	0.028	0.083	0.370
Poultry	−0.053	0.013	**0.305**	0.342

Note: The bolded numbers indicated that the dietary component was grouped to the corresponding dietary pattern.

**Table 2:** Baseline characteristics according to the trajectory of dietary patterns.

Characteristics	Southern Pattern			Rice-Vegetarian Pattern			Healthy Pattern			Alcohol-Meat Pattern		
	Low-Rapid Rise and Medium-Stable Group	High-Stable Group	*p*	Medium-Rapid Increase Group	High-Rapid Decline Group	*p*	Low-Stable Group	High-Rapid Increase Group	*p*	Low-Stable Group	Medium-High-Medium Group	*p*
*n*	389	660		845	204		1003	46		939	110	
Age (y)	41.7 ± 12.8	39.5 ± 12.2	0.007	40.7 ± 12.9	38.9 ± 10.2	0.065	40.1 ± 12.4	44.5 ± 12.6	0.019	40.4 ± 12.7	39.6 ± 10.6	0.528
Male, *n* (%)	183 (47.0)	327 (49.5)	0.472	383 (45.3)	127 (62.3)	<0.001	493 (49.2)	17 (37.0)	0.142	424 (45.2)	86 (78.2)	<0.001
Rural, *n* (%)	293 (75.3)	438 (66.4)	0.003	537 (63.6)	194 (95.1)	<0.001	724 (72.2)	7 (15.2)	<0.001	651 (69.3)	80 (72.7)	0.533
BMI (kg/m^2^)	21.7 ± 2.4	22.0 ± 2.9	0.156	22.0 ± 2.8	21.4 ± 2.4	0.007	21.9 ± 2.7	22.6 ± 3.1	0.083	21.9 ± 2.7	22.3 ± 2.6	0.146
SBP (mmHg)	109.0 ± 13.2	109.2 ± 11.5	0.839	109.1 ± 12.3	109.2 ± 11.1	0.944	109.1 ± 12.2	109.6 ± 10.7	0.807	108.9 ± 12.3	111.0 ± 10.1	0.109
DBP (mmHg)	71.2 ± 9.4	70.9 ± 8.7	0.712	70.9 ± 8.9	71.4 ± 8.9	0.538	71.0 ± 9.0	71.4 ± 8.2	0.782	70.8 ± 9.0	73.0 ± 8.3	0.023
Marital status, *n* (%)			0.393			0.074			0.510			0.280
Married	316 (81.2)	555 (84.1)		691 (81.8)	180 (88.2)		830 (82.8)	41 (89.1)		774 (82.4)	97 (88.2)	
Unmarried/divorced/widowed	72 (18.5)	102 (15.5)		151 (17.9)	23 (11.3)		169 (16.8)	5 (10.9)		161 (17.1)	13 (11.8)	
Missing	1 (0.3)	3 (0.5)		3 (0.4)	1 (0.5)		4 (0.4)	0 (0.0)		4 (0.4)	0 (0.0)	
Education, *n* (%)			0.001			<0.001			<0.001			0.003
Primary school or below	190 (48.8)	251 (38.0)		330 (39.1)	111 (54.4)		436 (43.5)	5 (10.9)		412 (43.9)	29 (26.4)	
Middle school	123 (31.6)	218 (33.0)		276 (32.7)	65 (31.9)		327 (32.6)	14 (30.4)		298 (31.7)	43 (39.1)	
High school or above	70 (18.0)	182 (27.6)		226 (26.7)	26 (12.7)		226 (22.5)	26 (56.5)		215 (22.9)	37 (33.6)	
Missing	6 (1.5)	9 (1.4)		13 (1.5)	2 (1.0)		14 (1.4)	1 (2.2)		14 (1.5)	1 (0.9)	
Smoking, *n* (%)	127 (32.6)	199 (30.2)	0.293	250 (29.6)	76 (37.3)	0.095	316 (31.5)	10 (21.7)	0.365	269 (28.6)	57 (51.8)	<0.001
Drinking, *n* (%)	187 (48.1)	251 (38.0)	0.001	340 (40.2)	98 (48.0)	0.119	420 (41.9)	18 (39.1)	0.845	359 (38.2)	79 (71.8)	<0.001
PAL, *n* (%)			0.01			<0.001			<0.001			<0.001
Light	107 (27.5)	234 (35.5)		324 (38.3)	17 (8.3)		303 (30.2)	38 (82.6)		312 (33.2)	29 (26.4)	
Moderate	58 (14.9)	116 (17.6)		162 (19.2)	12 (5.9)		168 (16.7)	6 (13.0)		137 (14.6)	37 (33.6)	
Vigorous	222 (57.1)	308 (46.7)		355 (42.0)	175 (85.8)		528 (52.6)	2 (4.3)		486 (51.8)	44 (40.0)	
Missing	2 (0.5)	2 (0.3)		4 (0.5)	0 (0.0)		4 (0.4)	0 (0.0)		4 (0.4)	0 (0.0)	
Diabetes	7 (1.8)	12 (1.8)	0.730	18 (2.1)	1 (0.5)	0.155	18 (1.8)	1 (2.2)	<0.001	19 (2.0)	0 (0.0)	0.237

Note: Values are presented as mean (SD) for continuous variables, and numbers (proportions) for categorical variables. The sums of percentages may not reach 100%, owing to the rounding of decimals and missing values. BMI, body mass index; SBP, systolic blood pressure; DPB, diastolic blood pressure; PAL, physical activity level.

**Table 3:** The associations of dietary pattern trajectories and the risks of hypertension.

Dietary Pattern Trajectories	Crude Model HR (95% CI)	Model 1 HR (95% CI)	Model 2 HR (95% CI)	Model 3 HR (95% CI)	Model 4 HR (95% CI)
Southern pattern					
Low-rapid rise and medium-stable group	1 (ref)	1 (ref)	1 (ref)	1 (ref)	1 (ref)
High-stable group	0.78 (0.65, 0.92)	0.85 (0.71, 1.01)	0.83 (0.69, 0.99)	0.81 (0.68, 0.98)	0.80 (0.67, 0.96)
Rice-vegetarian pattern					
Medium-rapid increase group	1 (ref)	1 (ref)	1 (ref)	1 (ref)	1 (ref)
High-rapid decline group	0.97 (0.78, 1.19)	1.03 (0.82, 1.29)	1.05 (0.84, 1.33)	1.06 (0.84, 1.33)	1.06 (0.85, 1.34)
Healthy pattern					
Low-stable group	1 (ref)	1 (ref)	1 (ref)	1 (ref)	1 (ref)
High-rapid increase group	0.76 (0.47, 1.23)	0.57 (0.35, 0.95)	0.57 (0.34, 0.96)	0.60 (0.36, 0.99)	0.58 (0.35, 0.97)
Alcohol-meat pattern					
Low-stable group	1 (ref)	1 (ref)	1 (ref)	1 (ref)	1 (ref)
Medium-high-medium group	1.46 (1.14, 1.87)	1.48 (1.15, 1.91)	1.53 (1.17, 1.99)	1.46 (1.12, 1.91)	1.48 (1.13, 1.94)

Note: Model 1 was adjusted for age (years, continuous), sex (male or female), residence (rural or urban), education level (primary school or below, middle school, high school or above), and marital status (married or unmarried). Model 2 was further adjusted for smoking status (yes or no), alcohol consumption (yes or no), physical activity level (light, moderate, or vigorous), and total energy intake (kcal/day, continuous) based on Model 1. Model 3 was additionally adjusted for diabetes status (yes or no), baseline BMI (kg/m^2^, continuous), and BMI change (kg/m^2^, continuous). Model 4 was further adjusted for changes in sodium intake (g/day, continuous) based on Model 3.

## Data Availability

The original contributions presented in this study are included in the article. Further inquiries can be directed to the corresponding authors.

## Supplementary Materials

The following supporting information can be downloaded at: https://www.mdpi.com/article/10.3390/nu18010039/s1. Supplementary Table S1: The BIC and AvePP of model and trajectories fitting; Supplementary Table S2: Fitting parameters of different dietary pattern trajectories; Supplementary Table S3: Dietary characteristics of the people under the southern pattern; Supplementary Table S4: Dietary characteristics of the people under the rice-vegetarian pattern; Supplementary Table S5: Dietary characteristics of the people under the healthy pattern; Supplementary Table S6: Dietary characteristics of the people under the alcohol-meat pattern; Supplementary Table S7: The sensitivity analysis of the association of the trajectory of dietary patterns and hypertension; Supplementary Figure S1: Scree plot of factor analysis of dietary pattern; Supplementary Figure S2: Normality test of the data in this study.

## Abbreviations

The following abbreviations are used in this manuscript:

CHNS	China Health and Nutrition Survey
SBP	systolic blood pressure
DBP	diastolic blood pressure
BMI	body mass index
SD	standard deviation
KMO	Kaiser-Meyer-Olkin
GBTM	group-based trajectory model
BIC	Bayesian Information Criterion
AvePP	average posterior probability
HR	hazard ratio
CI	confidence interval
