# Supplementary material for: Associations of the Trajectories of Dietary Pattern and Hypertension: Results from the CHNS Cohort
Source: Nutrients. 2025 Dec 22;18(1):39. doi: 10.3390/nu18010039 (PMC12787766; doi:10.3390/nu18010039)
Supplement: Supplementary file 1 [file nutrients-18-00039-s001.zip › nutrients-4015862-supplementary.pdf]

Supplementary Materials

Association of the trajectory of dietary patterns and hypertension in Chinese adults: A prospective cohort study

Hongxia Li[1,2,3#], Zhuangyu Zhang[4#], Die Shan[1, 2, 3], Zhiqiang Cao[1, 2, 3], Jingjing Li[5], Ling Liu[5], Yingying Ouyang[5], Chenrui Gong[5], Yuhan Tang[1, 2, 3], Ping Yao[1, 2, 3], Yi Song[5*], Shuang Liu[5*]

[1] Department of Nutrition and Food Hygiene, School of Public Health, Tongji Medical College, Huazhong University of Science and Technology, 13 Hangkong Rd, Wuhan 430030, China

[2] Hubei Key Laboratory of Food Nutrition and Safety, School of Public Health, Tongji Medical College, Huazhong University of Science and Technology, 13 Hangkong Rd, Wuhan 430030, China

[3] Experimental Teaching Center of Preventive Medicine, School of Public Health, Tongji Medical College, Huazhong University of Science & Technology

[4] Jiang'an County Health Bureau, Yibin 644299, China

[5] Institute of Health Surveillance Analysis and Protection, Hubei Provincial Center for Disease Control and Prevention, Wuhan 430079, China

[#] Co-first author: These authors contributed equally to this work and should be considered co-first authors.

* Corresponding author Shuang Liu: Email: ls_hbcdc@163.com and Yi Song : Email: hbcdc_songyi@163.com.

Supplementary Table S1 The BIC and AvePP of the model and trajectories fitting

Supplementary Table S2 Fitting parameters of different dietary pattern trajectories

Supplementary Table S3 Dietary characteristics of the people under the southern pattern

Supplementary Table S4 Dietary characteristics of the people under the rice-vegetarian pattern

Supplementary Table S5 Dietary characteristics of the people under the healthy pattern

Supplementary Table S6 Dietary characteristics of the people under the alcohol-meat pattern

Supplementary Table S7 The sensitivity analysis of the association of the trajectory of dietary patterns and hypertension

Supplementary Figure S1 Scree plot of factor analysis of dietary pattern

Supplementary Figure S2 Normality test of the data in this study

Supplementary Table S1 The BIC and AvePP of the model and trajectories fitting

Dietary pattern trajectories		BIC	Entropy	AvePP
**Southern pattern**	Low-rapid rise	-8512.6	0.79	0.96
	Medium-stable group			0.88
	High-stable group			0.91
**Rice-vegetarian pattern**	Medium-rapid increase group	-9004.4	0.71	0.93
	High-rapid decline group			0.84
**Healthy pattern**	Low-stable group	-7300.41	0.92	0.98
	High-rapid increase group			0.93
**Alcohol-meat pattern**	Low-stable group	-9329.13	0.88	0.98
	Medium-high-medium group			0.88

Supplementary Table S2 Fitting parameters of different dietary pattern trajectories

Trajectory types	Parameter	Coefficient	Standard error	*P*
**Southern pattern**				
Low-rapid rise	Intercept	2323.539	2.011	< 0.001
	Linear term	-2.369	0.301	< 0.001
	Squared term	0.001	0.001	< 0.001
Medium-stable group	Intercept	3119.361	1.271	< 0.001
	Linear term	-3.111	0.312	< 0.001
	Squared term	0.001	0.001	< 0.001
High-stable group	Intercept	-7.504	3.826	0.049
	Linear term	0.004	0.002	0.037
**Rice-vegetarian pattern**				
Medium-rapid increase group	Intercept	-8130.815	0.502	< 0.001
	Linear term	8.143	0.138	< 0.001
	Squared term	-0.002	0.001	< 0.001
High-rapid decline group	Intercept	176.725	8.790	< 0.001
	Linear term	-0.088	0.004	< 0.001
**Healthy pattern**				
Low-stable group	Intercept	-30.402	2.697	< 0.001
	Linear term	0.015	0.001	< 0.001
High-rapid increase group	Intercept	-10870.536	0.590	< 0.001
	Linear term	10.769	0.158	< 0.001
	Squared term	-0.003	0.001	< 0.001

**Alcohol-meat pattern**				
Low-stable group	Intercept	-22.069	3.648	< 0.001
	Linear term	0.011	0.002	< 0.001
Medium-high-medium group	Intercept	-33675.692	0.309	< 0.001
	Linear term	33.558	0.048	< 0.001
	Squared term	-0.008	0.001	< 0.001

Supplementary Table S3 Dietary characteristics of the people under the southern pattern

**Dietary characteristics**	**Southern pattern**		
	**Low-rapid rise & medium-stable group**	**High-stable group**	***P***
**Grain (g/d)**	518.6 (413.2, 615.4)	426.1 (349.4, 517.2)	< 0.001
**Whole grain (g/d)**	11.1 (0.0, 37.9)	5.5 (0.0, 16.7)	< 0.001
**Tubers (g/d)**	26.0 (8.3, 46.7)	16.7 (0.0, 36.7)	< 0.001
**Vegetables (g/d)**	406.9 (326.7, 508.1)	387.0 (314.8, 482.9)	0.082
**Dark-colored vegetables (g/d)**	79.9 (47.2, 132.2)	94.1 (61.6, 142.3)	0.001
**Fruit (g/d)**	0.0 (0.0, 0.0)	0.0 (0.0, 25.2)	< 0.001
**Legumes (g/d)**	6.9 (0.0, 15.4)	11.8 (4.8, 20.4)	< 0.001
**Aquatic products (g/d)**	1.3 (0.0, 22.0)	50.0 (23.3, 76.3)	< 0.001
**Poultry (g/d)**	0.0 (0.0, 8.9)	0.0 (0.0, 11.1)	0.244
**Eggs (g/d)**	13.3 (2.5, 27.3)	23.6 (12.3, 36.8)	< 0.001
**Nuts (g/d)**	0.0 (0.0, 5.6)	0.0 (0.0, 8.3)	0.005
**Red meat (g/d)**	32.7 (8.3, 59.0)	59.5 (28.9, 94.6)	< 0.001
**Oil (g/d)**	37.0 (27.1, 50.5)	38.4 (27.5, 49.8)	0.669
**Sodium (g/d)**	5.7 (4.3, 7.6)	5.1 (3.9, 6.6)	< 0.001
**Energy (kcal/d)**	2431.9 (2084.2, 2750.2)	2294.3 (2008.3, 2558.7)	< 0.001
**Protein (g/d)**	67.9 (59.5, 78.5)	68.6 (59.5, 78.4)	0.487
**Fat (g/d)**	65.1 (50.7, 80.4)	72.7 (56.9, 91.5)	< 0.001
**Carbohydrate (g/d)**	368.3 (300.6, 450.8)	318.1 (261.4, 382.7)	< 0.001

Data are presented as mean ± SD.

Supplementary Table S4 Dietary characteristics of the people under the rice-vegetarian pattern

**Dietary characteristics**	**Rice-vegetarian pattern**		
	**Medium-rapid increase group**	**High-rapid decline group**	***P***
**Grain (g/d)**	426.4 (357.6, 526.9)	569.5 (497.1, 640.3)	< 0.001
**Whole grain (g/d)**	8.3 (0.0, 23.8)	0.0 (0.0, 16.7)	0.003
**Tubers (g/d)**	20.8 (0.0, 39.4)	23.6 (0.0, 45.9)	0.314
**Vegetables (g/d)**	369.9 (305.2, 445.8)	530.5 (454.1, 627.2)	< 0.001
**Dark-colored vegetables (g/d)**	83.3 (52.5, 125.0)	131.2 (80.0, 191.2)	< 0.001
**Fruit (g/d)**	0.0 (0.0, 16.7)	0.0 (0.0, 6.8)	0.083
**Legumes (g/d)**	11.0 (3.8, 19.3)	6.2 (1.4, 16.0)	< 0.001
**Aquatic products (g/d)**	30.7 (0.0, 59.3)	33.3 (0.0, 77.8)	0.015
**Poultry (g/d)**	0.0 (0.0, 11.1)	0.0 (0.0, 4.9)	0.016
**Eggs (g/d)**	21.7 (10.0, 35.3)	14.2 (5.6, 25.3)	< 0.001
**Nuts (g/d)**	0.0 (0.0, 6.7)	0.0 (0.0, 11.1)	0.018
**Red meat (g/d)**	54.4 (24.3, 87.5)	28.1 (6.0, 57.0)	< 0.001
**Oil (g/d)**	38.4 (27.2, 51.0)	36.5 (27.6, 45.7)	0.096
**Sodium (g/d)**	5.2 (3.9, 6.9)	5.8 (4.8, 7.3)	0.001
**Energy (kcal/d)**	2268.9 (1981.8, 2559.6)	2589.2 (2370.2, 2856.2)	< 0.001
**Protein (g/d)**	67.4 (58.4, 77.6)	72.9 (64.3, 81.7)	< 0.001
**Fat (g/d)**	71.7 (55.7, 90.1)	64.3 (50.0, 78.5)	< 0.001
**Carbohydrate (g/d)**	315.4 (261.9, 383.4)	417.9 (364.6, 467.7)	< 0.001

Data are presented as mean ± SD.

Supplementary Table S5 Dietary characteristics of the people under the healthy pattern

Dietary characteristics	Healthy pattern		
	Low-stable group	High-rapid increase group	*P*
**Grain (g/d)**	466.7 (379.0, 567.5)	343.4 (291.5, 409.1)	<0.001
**Whole grain (g/d)**	6.6 (0.0, 23.8)	4.7 (0.0, 14.6)	0.25
**Tubers (g/d)**	21.1 (0.0, 41.6)	20.4 (12.7, 35.8)	0.637
**Vegetables (g/d)**	396.0 (319.1, 490.5)	392.7 (296.6, 481.6)	0.332
**Dark-colored vegetables (g/d)**	88.9 (55.3, 138.3)	82.8 (61.9, 118.3)	0.831
**Fruit (g/d)**	0.0 (0.0, 10.0)	79.3 (7.4, 150.9)	<0.001
**Legumes (g/d)**	10.1 (3.1, 18.1)	14.3 (7.3, 25.6)	0.018
**Aquatic products (g/d)**	30.0 (0.0, 61.7)	49.8 (29.9, 66.5)	0.003
**Poultry (g/d)**	0.0 (0.0, 8.3)	16.7 (5.7, 31.9)	<0.001
**Eggs (g/d)**	19.0 (8.3, 33.1)	38.4 (22.9, 50.4)	<0.001
**Nuts (g/d)**	0.0 (0.0, 6.7)	5.3 (0.0, 25.0)	<0.001
**Red meat (g/d)**	45.4 (18.6, 77.6)	108.5 (76.2, 134.2)	<0.001
**Oil (g/d)**	37.9 (27.2, 50.0)	40.3 (33.2, 47.3)	0.277
**Sodium (g/d)**	5.4 (4.1, 6.9)	4.0 (2.9, 5.3)	<0.001
**Energy (kcal/d)**	2336.8 (2050.2, 2630.3)	2258.1 (1936.8, 2563.4)	0.413
**Protein (g/d)**	68.0 (59.2, 77.7)	80.9 (65.1, 89.5)	<0.001
**Fat (g/d)**	69.2 (53.9, 86.9)	86.8 (70.1, 102.2)	<0.001
**Carbohydrate (g/d)**	342.6 (275.8, 412.6)	275.3 (243.8, 316.3)	<0.001

Data are presented as mean ± SD.

Supplementary Table S6 Dietary characteristics of the people under the alcohol-meat pattern

Dietary characteristics	Alcohol-meat pattern		
	Low-stable group	Medium-high-medium group	*P*
**Grain (g/d)**	466.7 (375.0, 566.7)	415.0 (360.6, 517.7)	0.021
**Whole grain (g/d)**	6.7 (0.0, 23.8)	1.2 (0.0, 16.7)	0.117
**Tubers (g/d)**	21.4 (0.0, 42.2)	16.7 (0.0, 32.6)	0.061
**Vegetables (g/d)**	396.7 (316.7, 492.6)	381.0 (320.2, 466.9)	0.495
**Dark-colored vegetables (g/d)**	88.9 (55.1, 137.5)	89.3 (62.0, 136.4)	0.771
**Fruit (g/d)**	0.0 (0.0, 11.6)	0.0 (0.0, 23.2)	0.019
**Legumes (g/d)**	9.8 (2.7, 17.4)	17.6 (9.4, 27.0)	<0.001
**Aquatic products (g/d)**	29.2 (0.0, 59.2)	50.0 (16.7, 77.7)	<0.001
**Poultry (g/d)**	0.0 (0.0, 8.3)	6.9 (0.0, 27.8)	<0.001
**Eggs (g/d)**	19.5 (8.3, 33.3)	23.0 (13.1, 37.4)	0.055
**Nuts (g/d)**	0.0 (0.0, 6.7)	0.0 (0.0, 9.9)	0.298
**Red meat (g/d)**	44.3 (16.9, 75.0)	99.7 (50.4, 132.1)	<0.001
**Oil (g/d)**	37.4 (27.2, 49.5)	42.5 (31.0, 55.8)	0.005
**Sodium (g/d)**	5.2 (4.0, 6.7)	6.6 (5.0, 9.0)	<0.001
**Energy (kcal/d)**	2303.6 (2015.4, 2594.6)	2593.1 (2334.6, 2936.3)	<0.001
**Protein (g/d)**	67.2 (58.8, 76.2)	80.5 (72.4, 92.7)	<0.001
**Fat (g/d)**	68.4 (53.3, 85.2)	89.8 (65.9, 105.7)	<0.001
**Carbohydrate (g/d)**	340.3 (273.0, 409.5)	324.3 (276.4, 399.2)	0.585

Data are presented as mean ± SD.

Supplementary Table S7 The sensitivity analysis of the association of the trajectory of dietary patterns and hypertension

Sensitivity analysis	HR (95% CI)			
	Southern pattern[a]	Rice-vegetarian pattern[b]	Healthy pattern[c]	Alcohol-meat pattern[d]
Exclude those with diabetes, stroke, or myocardial infarction at baseline				
Crude model	0.78 (0.65, 0.93)	0.97 (0.79, 1.20)	0.72 (0.44, 1.19)	1.48 (1.16, 1.90)
Model 1	0.86 (0.72, 1.02)	1.01 (0.81, 1.26)	0.55 (0.32, 0.92)	1.48 (1.15, 1.91)
Model 2	0.83 (0.69, 0.99)	1.04 (0.83, 1.31)	0.54 (0.32, 0.92)	1.53 (1.17, 1.99)
Model 3	0.82 (0.68, 0.98)	1.04 (0.82, 1.31)	0.55 (0.33, 0.94)	1.46 (1.12, 1.91)
Model 4	0.81 (0.67, 0.97)	1.04 (0.83, 1.32)	0.54 (0.31, 0.91)	1.48 (1.13, 1.93)
Multiple imputation				
Crude model	0.78 (0.65, 0.92)	0.97 (0.78,1.19)	0.76 (0.47,1.23)	1.46 (1.14,1.87)
Model 1	0.85 (0.71, 1.01)	1.02 (0.82, 1.28)	0.57 (0.34, 0.95)	1.47 (1.14, 1.90)
Model 2	0.84 (0.70, 1.01)	1.05 (0.83, 1.31)	0.59 (0.35, 0.98)	1.53 (1.17, 1.99)
Model 3	0.84 (0.69, 1.01)	1.06 (0.84, 1.33)	0.62 (0.37, 1.03)	1.44 (1.10, 1.88)
Model 4	0.83 (0.69, 0.99)	1.07 (0.85, 1.34)	0.60 (0.36, 0.99)	1.45 (1.11, 1.90)
Exclude those with missing covariates				
Crude model	0.75 (0.62, 0.91)	0.93 (0.74, 1.17)	0.86 (0.52, 1.41)	1.40 (1.07, 1.84)
Model 1	0.84 (0.67, 1.02)	0.99 (0.77, 1.27)	0.61 (0.36, 1.04)	1.36 (1.03, 1.80)
Model 2	0.80 (0.65, 0.98)	1.05 (0.81, 1.36)	0.62 (0.37, 1.06)	1.42 (1.06, 1.90)
Model 3	0.78 (0.64, 0.97)	1.04 (0.80, 1.35)	0.60 (0.35, 1.03)	1.36 (1.01, 1.82)
Model 4	0.77 (0.62, 0.95)	1.05 (0.81, 1.36)	0.58 (0.34, 0.98)	1.37 (1.02, 1.84)

Note: Model 1 was adjusted for age (years, continuous), sex (male or female), residence (rural or urban), education level (primary school or below, middle school, high school or above), and marital status (married or unmarried).

Model 2 was further adjusted for smoking status (yes or no), alcohol consumption (yes or no), physical activity level (light, moderate, or vigorous), and total energy intake (kcal/day, continuous) based on Model 1.

Model 3 was additionally adjusted for diabetes status (yes or no), baseline BMI (kg/m^2, continuous), and BMI change (kg/m^2, continuous).

Model 4 was further adjusted for changes in sodium intake (g/day, continuous) based on Model 3.

[a]Southern pattern: The Low-rapid rise & medium-stable group as the reference.

[b]Rice-vegetarian pattern: The Medium-rapid increase group as the reference.

[c]Healthy pattern: Low-stable group as the reference.

[d]Alcohol-meat pattern: Low-stable group as the reference.

HR: hazard ratio; CI: confidence interval.

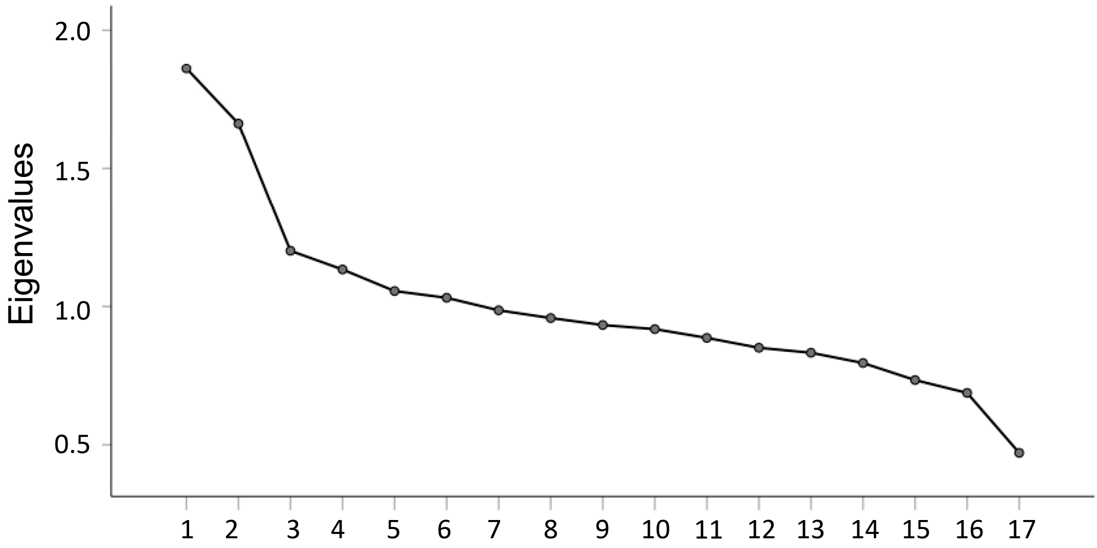

Supplementary Figure S1 Scree plot of factor analysis of dietary pattern.

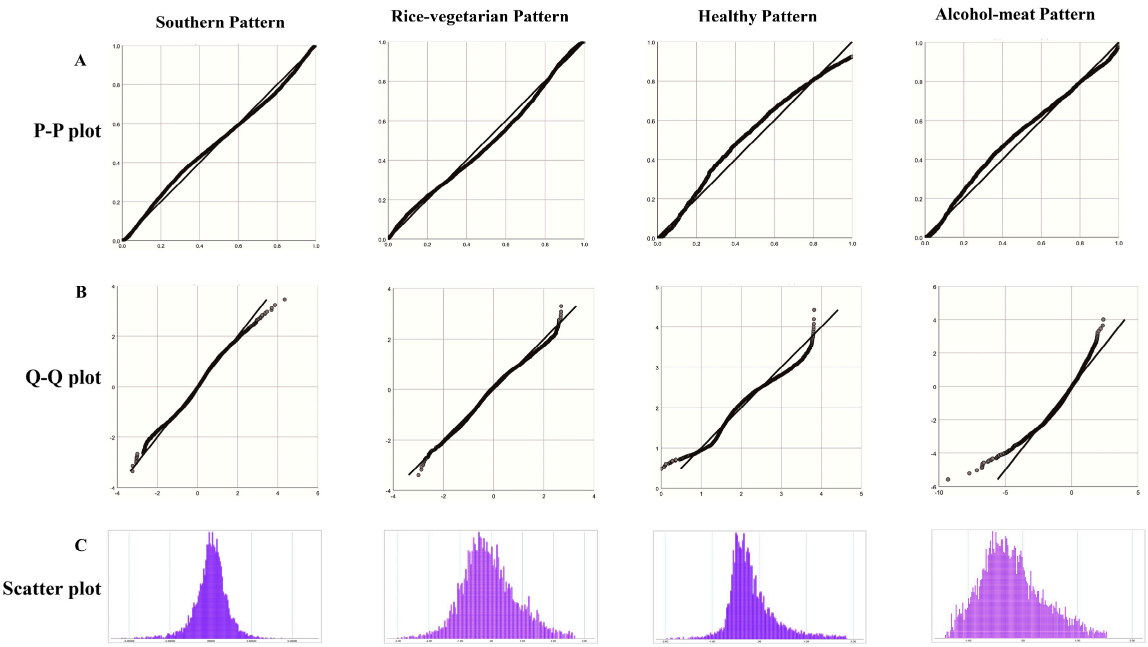

Supplementary Figure S2 Normality test of the data in this study. (A) The P-P plot of four dietary patterns. (B) The Q-Q plot of four dietary patterns. (C) The scatter plot of the data of the four dietary patterns.
